# Immunogenicity and safety of RAZI recombinant spike protein vaccine (RCP) as a booster dose after priming with BBIBP-CorV: a parallel two groups, randomized, double blind trial

**DOI:** 10.1186/s12916-024-03295-1

**Published:** 2024-02-20

**Authors:** Saeed Erfanpoor, Seyed Reza Banihashemi, Ladan Mokhbaeralsafa, Saeed Kalantari, Ali Es-haghi, Mojtaba Nofeli, Ali Rezaei Mokarram, Fariba Sadeghi, Monireh Hajimoradi, Seyad Hossein Razaz, Maryam Taghdiri, Mohsen Lotfi, Akbar Khorasani, Akram Ansarifar, Safdar Masoumi, Arash Mohazzab, Sara Filsoof, Vahideh Mohseni, Masoumeh Shahsavan, Niloufar Gharavi, Seyed Amin Setarehdan, Mohammad Hasan Rabiee, Mohammad Hossein Fallah Mehrabadi, Masoud Solaymani-Dodaran

**Affiliations:** 1 School of Public Health, Department of Epidemiology, Iran University of Medical Science, Tehran, Iran; 2 Department of Immunology, Agricultural Research, Education and Extension Organization (AREEO), Razi Vaccine and Serum Research Institute, Karaj, Iran; 3 Department of Epidemiology, Razi Vaccine and Serum Research Institute, Agricultural Research, Education and Extension Organization (AREEO), Karaj, Iran; 4 Departments of Infectious Diseases and Tropical Medicine, Iran University of Medical Sciences, Tehran, Iran; 5 Department of Physico Chemistry, Razi Vaccine and Serum Research Institute, Agricultural Research, Education and Extension Organization (AREEO), Karaj, Iran; 6 Department of Research and Development, Razi Vaccine and Serum Research Institute, Agricultural Research, Education and Extension Organization (AREEO), Karaj, Iran; 7 Department of QA, Razi Vaccine and Serum Research Institute, Agricultural Research, Education and Extension Organization (AREEO), Karaj, Iran; 8 Department of Quality Control, Razi Vaccine and Serum Research Institute, Agricultural Research, Education and Extension Organization (AREEO), Karaj, Iran; 9 Department of Biostatistics, Faculty of Medical Sciences, Tarbiat Modares University, Tehran, Iran; 10 Reproductive Biotechnology Research Center, Avicenna Research Institute Tehran, ACECR, Tehran, Iran; 11 School of Medicine, Iran University of Medical Science, Tehran, Iran; 12 Minimally Invasive Surgery Research Center, Hazrat-E-Rasool Hospital, Iran University of Medical Science, Tehran, Iran; 13 Division of Epidemiology, Faculty of Veterinary Medicine, University of Tehran, Tehran, Iran; 14 Clinical Trial Center, Iran University of Medical Science, Tehran, Iran; 15 Division of Epidemiology and Public Health, University of Nottingham, Nottingham, NG7 2UH UK

**Keywords:** SARS-CoV-2, Recombinant Vaccine, Booster dose, Heterologous boosting

## Abstract

**Background:**

The immunity induced by primary vaccination is effective against COVID-19; however, booster vaccines are needed to maintain vaccine-induced immunity and improve protection against emerging variants. Heterologous boosting is believed to result in more robust immune responses. This study investigated the safety and immunogenicity of the Razi Cov Pars vaccine (RCP) as a heterologous booster dose in people primed with Beijing Bio-Institute of Biological Products Coronavirus Vaccine (BBIBP-CorV).

**Methods:**

We conducted a randomized, double-blind, active-controlled trial in adults aged 18 and over primarily vaccinated with BBIBP-CorV, an inactivated SARS-CoV-2 vaccine. Eligible participants were randomly assigned (1:1) to receive a booster dose of RCP or BBIBP-CorV vaccines. The primary outcome was neutralizing antibody activity measured by a conventional virus neutralization test (cVNT). The secondary efficacy outcomes included specific IgG antibodies against SARS-CoV-2 spike (S1 and receptor-binding domain, RBD) antigens and cell-mediated immunity. We measured humoral antibody responses at 2 weeks (in all participants) and 3 and 6 months (a subgroup of 101 participants) after the booster dose injection. The secondary safety outcomes were solicited and unsolicited immediate, local, and systemic adverse reactions.

**Results:**

We recruited 483 eligible participants between December 7, 2021, and January 13, 2022. The mean age was 51.9 years, and 68.1% were men. Neutralizing antibody titers increased about 3 (geometric mean fold increase, GMFI = 2.77, 95% CI 2.26–3.39) and 21 (GMFI = 21.51, 95% CI 16.35–28.32) times compared to the baseline in the BBIBP-CorV and the RCP vaccine groups. Geometric mean ratios (GMR) and 95% CI for serum neutralizing antibody titers for RCP compared with BBIBP-CorV on days 14, 90, and 180 were 6.81 (5.32–8.72), 1.77 (1.15–2.72), and 2.37 (1.62–3.47) respectively. We observed a similar pattern for specific antibody responses against S1 and RBD. We detected a rise in gamma interferon (IFN-γ), tumor necrosis factor (TNF-α), and interleukin 2 (IL-2) following stimulation with S antigen, particularly in the RCP group, and the flow cytometry examination showed an increase in the percentage of CD3 + /CD8 + lymphocytes. RCP and BBIBP-CorV had similar safety profiles; we identified no vaccine-related or unrelated deaths.

**Conclusions:**

BBIBP-CorV and RCP vaccines as booster doses are safe and provide a strong immune response that is more robust when the RCP vaccine is used. Heterologous vaccines are preferred as booster doses.

**Trial registration:**

This study was registered with the Iranian Registry of Clinical Trial at www.irct.ir, IRCT20201214049709N4. Registered 29 November 2021.

**Supplementary Information:**

The online version contains supplementary material available at 10.1186/s12916-024-03295-1.

## Background

The World Health Organization declared SARS-CoV-2 pandemic on 11 March 2020 [1]. Globally, until June 2023, more than 767 million confirmed cases of COVID-19 have been recorded, and nearly 7 million people have died due to this disease [2]. The COVID-19 pandemic has led to global efforts to develop safe and effective vaccines against the rapidly spreading virus [3, 4]. The COVID-19 vaccination programs have effectively protected against severe disease, hospitalization, and death [5, 6]. Protection against SARS-CoV-2 infection caused by primary vaccination wanes significantly over time [7], and this is more alarming considering the continuous emergence of new SARS-CoV-2 strains [8]. Regardless of the type and platform of COVID-19 vaccines, their effectiveness decreases after 3 to 6 months. It has been reported that following a complete vaccination, the effectiveness of the BNT162b2 vaccine against COVID-19 infection decreased from 90.8% in the early period (first 2 months) to 79.3% in the late period (3 to 5 months later). The corresponding figures of vaccine effectiveness for the CoronaVac vaccine were 74.5% and 30.4% [9].

People who received a booster dose (either homologous or heterologous) had more robust immune responses and less severe illness or infection with COVID-19 than those that did not receive it, regardless of the type of the primary vaccine [10, 11]. Furthermore, there are reports that a heterologous boosting may provide additional immunity and protection against SARS-CoV-2 infection variants [1, 12–14]. Adminstration of viral vector, mRNA, or recombinant protein-based vaccines in individuals with a history of two doses of inactivated vaccine has resulted in strong immunogenicity with acceptable adverse events [1, 13, 15]. There are also reports that heterologous boosting by recombinant subunit vaccines, such as NVSI-06–07, V-01, ZF2001, and SpikoGen®, in individuals primed with two doses of inactivated vaccines is immunogenically superior to homologous boosting [15–18].

RAZI Cov Pars (RCP) is a recombinant spike protein COVID-19 vaccine developed by the Razi Vaccine and Serum Research Institute of Iran. It comprises three components of monomeric S1 (amino acid 1–674), S2 (amino acid 685–1211) subunits, and trimeric S protein formulated in an oil-in-water adjuvant system RAS-01 (Razi Adjuvant System-01). A detailed description of RCP preparation has been published before [19]. RCP has shown promising safety and induced robust and long-lasting humoral and cellular immune responses in preclinical and all three phases of clinical trials [20–22] (phase III trial results are under publication). Sinopharm inactivated virus vaccine (BBIBP-CorV), which the World Health Organization approves, has been widely used in many countries, including Iran’s vaccination program (about 70% of the coverage) [23]. Based on the above, using a recombinant protein sub-unit such as RCP is an appropriate choice of booster vaccine in the face of declining immunity [24–26] following primary vaccination with BBIBP-CorV. The current study explores the safety and immunogenicity of heterologous boosting with RCP compared to homologous boosting with BBIBP-CorV in adults who have previously received two doses of the inactivated BBIBP-CorV.

## Methods

### Study design

We conducted a multicenter, randomized, double-blind, parallel-group, active control trial in adults 18 and older who were vaccinated primarily with an inactivated SARS-CoV-2 vaccine-BBIBP-CorV. Participants were randomly assigned (1:1) to receive a booster dose of RCP or BBIBP-CorV vaccine in the two trial centers. The study protocol was approved by the National Research Ethics Committee (approval number IR.NREC.1400.013) and registered with the Iranian Registry of Clinical Trial (Ref: IRCT20201214049709N4).

### Participants

Participants were adult Iranian nationals or legal residents aged 18 years or older who had completed their primary vaccination with two doses of BBIBP-CorV vaccine at least 75 days and at most 195 days before their enrollment. The main exclusion criteria were as follows: history of allergic diseases such as angioedema or anaphylactic reactions after receiving previous COVID-19 vaccines; any current or new diagnosis of acute or chronic illness requiring continuous ongoing medical care; pregnancy and lactation; immunodeficiency diseases (suspected and definitive); history of uncontrolled serious psychiatric illnesses; history of blood disorders (dyscrasia, coagulopathy, platelet deficiency or disorder, or deficiency of blood clotting factors); history of chronic neurological diseases (including seizures and epilepsy) and acute febrile illness at the time of booster vaccine injection.

### Randomization and masking

A stratified block randomization method with a block size of 4 was used to assign each participant to the intervention groups. Stratification was based on four-time strata of 2.5–3.5, 3.5–4.5, 4.5–5.5, and 5.5–6.5 months passed from the participants’ primary vaccination. The rand () function of Excel software was used to generate a random sequence within each block. A non-repetitive five-digit random code was used to conceal the chain of randomization. In this study, the BBIBP-CorV vaccine had different packaging and shape than RCP. Once the participant reached the vaccine injection stage, the assigned vaccine type was temporarily displayed on the computer screen and disappeared following the confirmation of the injection. Therefore, the person responsible for injecting the vaccine was the only research team member who was aware of the intervention type and trained not to disclose this to others.

### Procedures

We enrolled volunteers via a website. Those who successfully passed the online screening were invited to attend the two trial centers. Potential participants were asked to sign a written informed consent and further evaluated for eligibility. Eligible participants randomly received an intramuscular dose of either 10 μg/200 μL RAZI recombinant spike protein (RCP) or 0.5 ml Sinopharm inactivated virus (BBIBP-Corv) vaccines. Blood samples for immunogenicity were collected from the participants at the time of booster vaccination and on days 14, 90, and 180 after that.

Participants were monitored for half an hour after receiving the injection for acute anaphylactic reactions. They were asked to report their local (pain, tenderness, erythema/redness, and swelling) and systemic (nausea, diarrhea, headache, fatigue, and myalgia) adverse reactions every day for 7 days via an application installed on their mobile phone. A 24/7 follow-up center with a resident physician could be contacted by phone during the 1-month follow-up period. Participants had to report weekly any visit to a medical center or medication use through their mobile application; otherwise, they were flagged for active follow-up by the research team.

### Outcomes

The primary outcome was neutralizing antibody titer on days 14, 90, and 180 following the injection measured by conventional virus neutralization test (cVNT). The test was conducted in a biosafety level 3 laboratory facility using the original live alpha SARS-CoV-2 strain isolated from the Iranian COVID-19 patients. We defined seroconversion as a four-fold or more increase in the antibody titer compared to the baseline (day 0). Secondary efficacy outcomes were specific IgG antibody levels against S1 and RBD spike antigens of SARS-CoV-2 measured by ELISA on days 14, 90, and 180 and cellular immunity on day 14. We measured specific IgG levels in six serum dilutions (0·1, 0·01, 0·001, 0·0001, 0·00001, and 0·000001) for each specimen and estimated the response by calculating the area under the curve (AUC). Peripheral blood mononuclear cell cultures were used to assess the cellular immunity responses. We tested them for specific cytokine-secreting T cells before and after the stimulation by specific COVID-19 S1 antigens. The secretion of gamma interferon (IFN-γ), tumor necrosis factor (TNF-α), and interleukin IL-2, 4, 6, and 17 were detected by enzyme-linked immunosorbent assay (ELISA) (R&D, USA). CD3, CD4, CD8, CD3/CD8, and CD3/CD4 cell counts were analyzed by flow cytometer (BD FACSLyric, USA). Lymphocyte proliferative potential response was measured by CFSE (carboxyfluorescein succinimidyl ester) cell staining assay. For more details, please see Additional file 1 [21, 27].

Secondary safety outcomes were abnormal vital signs and anaphylactic reactions immediately after the vaccination, solicited local and systemic adverse reactions, medically attended adverse events, and serious adverse events up to 1 month after receiving the booster dose. We used FDA toxicity grading scales to classified solicited local and systemic adverse reactions [28]. We assessed the causality for the detected adverse events during the one-month follow-up period [29].

### Statistical analysis

All participants who underwent randomization were included in the safety population. The immunogenicity population consisted of all randomized participants who provided at least one serum sample after receiving the booster dose (modified intention to treat approach). Means and proportions were used to summarize demographic characteristics. Baseline comparisons were performed to examine the homogeneity between the study groups. Geometric mean for neutralizing antibody titers (GMT) and specific IgG antibody responses (area under the curve, AUC) were calculated. Geometric mean ratios (GMR) and their 95% confidence interval for RCP compared to BBIBP-CorV were estimated at different time intervals based on Dunnett’s test, which is used to adjust multiple comparisons with one control. Geometric mean fold increases (GMFI) were calculated for each vaccine by dividing the geometric mean response by that of the baseline. The data was analyzed by Stata 14.2 (Stata Corporation, Texas, USA).

## Results

### Participant characteristics and baseline comparison

**Fig. 1:**
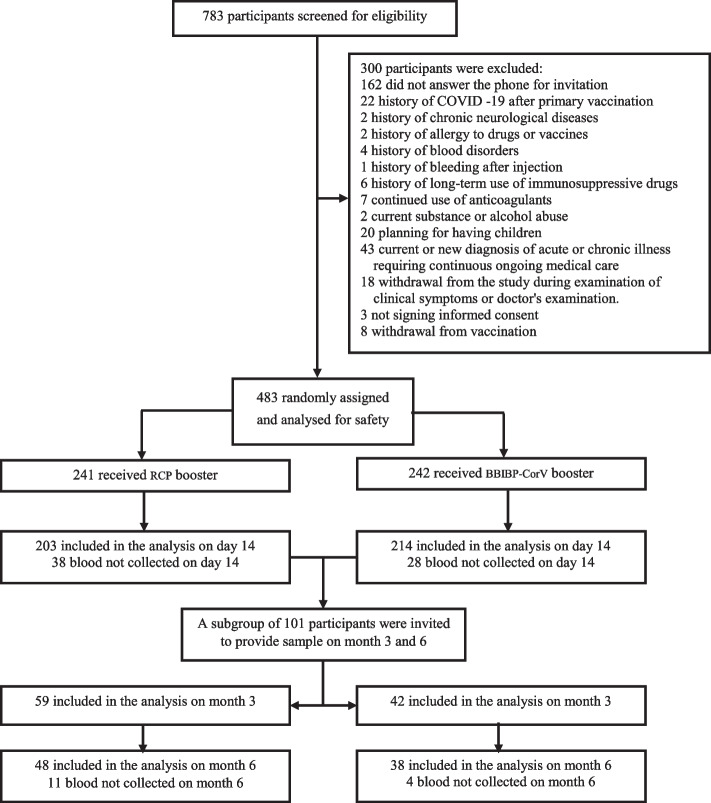
Participant flow diagram. Randomization and analysis populations. A total of 783 participants were enrolled, and 483 received booster vaccinations. The participants were randomly assigned to receive a booster dose of either RCP or BBIBP-CorV. All the 483 participants receiving booster vaccination were included in the safety set for safety analysis. We evaluated the immunogenicity in 417 participants who visited the research center for blood sampling on day 14 and a subgroup of 101 participants on 3 and 6 months after the booster dose

Between December 7, 2021, and January 13, 2022, 483 eligible participants randomly received BBIBP-CorV (241) or RCP (242) vaccines (Fig. 1) and followed for 7260 and 7207 person-days, respectively. The mean age of the participants in the study was 51.9 years, and 68.1% were men. A comparison of baseline characteristics indicates a balanced distribution of participants in the study groups (Table 1).

**Table 1:** A Comparison of baseline characteristics of the participant

	BBIBP-CorV *n* = 242	RCP *n* = 241	Total *n* = 483
Sex, *n* (%)			
Male	162 (66.9)	167 (69.3)	329 (68.1)
Female	80 (33.1)	74 (30.7)	154 (31.9)
Age, years			
Mean (SD)	51.94 (14.5)	51.95 (13.3)	51.94 (13.9)
Age group, *n* (%)			
18–28	12 (5.0)	15 (6.2)	27 (5.6)
28–38	37 (15.3)	30 (12.5)	67 (13.9)
38–48	44 (18.2)	40 (16.6)	84 (17.4)
48–58	63 (26.0)	69 (28.6)	132 (27.3)
58–68	70 (28.9)	69 (28.6)	139 (28.8)
> 68	15 (6.2)	18 (7.5)	33 (6.8)
Body mass index			
Mean (SD)	27.09 (4.0)	27.42 (4.3)	27.25 (4.2)
Education, *n* (%)			
No formal education	26 (10.7)	17 (7.1)	43 (8.9)
Up to diploma	62 (25.6)	72 (29.9)	134 (27.7)
Diploma	58 (24.0)	69 (28.6)	127 (26.3)
Diploma plus	26 (10.7)	11 (4.6)	37 (7.7)
Bachelor	44 (18.2)	47 (19.5)	91 (18.8)
Master	23 (9.5)	15 (6.2)	38 (7.9)
Doctoral and above	3 (1.2)	10 (4.2)	13 (2.7)
Comorbidities *n* (%)			
Hypertension	36 (14.9)	38 (15.8)	74 (15.3)
Chronic heart diseases	5 (2.1)	5 (2.1)	10 (2.1)
Chronic non-asthma lung diseases	0 (0.0)	2 (0.8)	2 (0.4)
Asthma	4 (1.7)	3 (1.2)	7 (1.5)
Chronic kidney diseases	1 (0.4)	0 (0.0)	1 (0.2)
Moderate or severe liver diseases	1 (0.4)	1 (0.4)	2 (0.4)
Mild liver diseases (fatty liver)	10 (4.1)	13 (5.4)	23 (4.8)
Chronic neurological diseases	3 (1.2)	4 (1.7)	7 (1.5)
Diabetes	21 (8.7)	25 (10.4)	46 (9.5)
Diabetes with complications	0 (0.0)	1 (0.4)	1 (0.2)
Chronic blood diseases	0 (0.0)	1 (0.4)	1 (0.2)
Rheumatic diseases	1 (0.4)	0 (0.0)	1 (0.2)
Dementia	0 (0.0)	0 (0.0)	0 (0.0)

### Immunogenicity

#### Humoral immune response

**Fig. 2:**
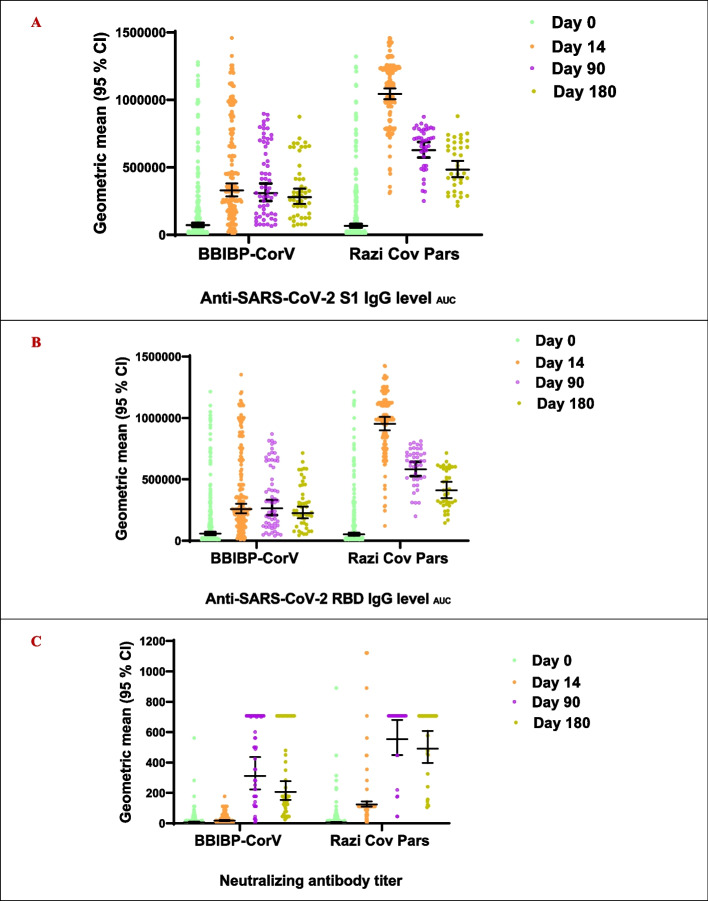
Geometric mean and 95% CI of specific antibody responses (AUC) to **A** S1, **B** RBD, and **C** neutralizing antibody titer in the BBIBP-CorV and RCP groups over the predefined study time schedule. Error bars are 95% CIs

Neutralizing antibody response 2 weeks after the booster dose (day 14) was statistically significantly higher in the RCP group compared with BBIBP-CorV (GMR = 6.8, 95% CI 5.3–8.7). The geometric mean of neutralizing antibody titers statistically significantly increased about 3 and 21 times the baseline in the BBIBP-CorV (GMFI = 2.8, 95% CI 2.2–3.3) and RCP (GMFI = 21.5, 95% CI 16.3–28.3) groups. The neutralizing antibody response remained high 3 and 6 months after the booster dose in both RCP and BBIBP-CorV groups (Table 2, Fig. 2C, and Additional file 1: Table S1). Similarly, specific antibody responses against S1 and RBD antigens on day 14 were statistically significantly higher in the RCP group compared with BBIBP-CorV (GMR = 3.1, 95% CI 2.7–3.7 and GMR = 3.6, 95% CI 3.1–4.3). The geometric mean of specific antibody response on day 14 increased about 4.4 and 15.7 times the baseline against S1 antigen and 4.3 and 18.2 times the baseline against RBD antigen in the BBIBP-CorV and RCP groups, respectively, and the increases were statistically significant. The specific antibody responses against S1 and RBD antigens gradually decreased over the next 6 months but remained 4 and 7 times the baseline level in the BBIBP-CorV and RCP groups (Table 2, Fig. 2A, B, and Additional file 1: Table S1). The baseline antibody levels and the antibody responses were similar across the time strata used for the randomization (Additional file 1: Table S2-S4 and Figure S2).

**Table 2:** Geometric mean ratio, geometric mean fold increase, and seroconversion and 95% CI of specific antibody responses (AUC) to S1, RBD, and neutralizing antibody titer in the BBIBP-CorV and RCP groups over the predefined study time schedule

	Neutralizing antibody titer^a^		Anti-SARS-CoV-2 S1 IgG level^b^_AUC_^c^		Anti-SARS-CoV-2 RBD IgG level_AUC_	
	BBIBP-CorV	RCP	BBIBP-CorV	RCP	BBIBP-CorV	RCP
GMFI (95% CI)^e^						
Baseline	Ref	Ref	Ref	Ref	Ref	Ref
Day 14	2.77 (2.26–3.39)	21.51 (16.35–28.32)	4.41 (3.66–5.32)	15.66 (12.58–19.16)	4.33 (3.58–5.25)	18.22 (14.66–22.64)
Day 90	48.62 (29.27–80.77)	90.22 (48.98–166.18)	4.92 (3.27–7.44)	8.37 (4.86–13.20)	5.39 (3.55–8.18)	9.34 (5.65–15.45)
Day 180	28.02 (17.18–45.72)	83.08 (45.75–150.91)	4.18 (2.74–6.37)	7.05 (4.13–12.04)	4.35 (2.84–6.66)	7.23 (4.17–12.51)
GMR (95% CI)^f^						
Baseline	Ref	0.88 (0.63–1.22)	Ref	0.92 (0.70–1.21)	Ref	0.90 (0.68–1.19)
Day 14	Ref	6.81 (5.32–8.72)	Ref	3.17 (2.73–3.70)	Ref	3.67 (3.14–4.30)
Day 90	Ref	1.77 (1.15–2.72)	Ref	2.03 (1.57–2.64)	Ref	2.20 (1.65–2.94)
Day 180	Ref	2.37 (1.62–3.47)	Ref	1.73 (1.35–2.21)	Ref	1.81 (1.38–2.39)
Seroconversion, *n*/*N* (95% CI)^g^						
Day 14	69/211, 33% (26–39)	157/202, 78% (71–83)	99/202, 49% (42–56)	152/195, 78% (72–83)	100/202, 49% (42–56)	157/195, 80% (74–850)
Day 90	55/59, 93% (84–98)	39/42, 93% (80–98)	29/59, 49% (36–61)	25/41, 60% (45–75)	32/59, 54% (41–66)	25/41, 60% (44–75)
Day 180	42/48, 87% (75–95)	36/37, 97% (85–99)	24/48, 50% (36–64)	24/38, 63% (48–78)	25/48, 52% (38–66)	23/38, 61% (45–76)

The number of participants at each time point may be different due to spoilage of blood samples in the laboratory and withdrawal from the study

^a^Were assessed via a conventional virus neutralization test

^b^Were measured using house ELISA kits and specific COVID-19 antigens (Native Antigen, UK)

^c^Area under the curve

^d^Geometric mean

^e^Geometric mean fold increase

^f^Geometric mean ratio

^g^4-fold increase compared to the baseline in neutralizing antibody titer or specific IgG antibody level (AUC)

#### Cellular immune response

**Fig. 3:**
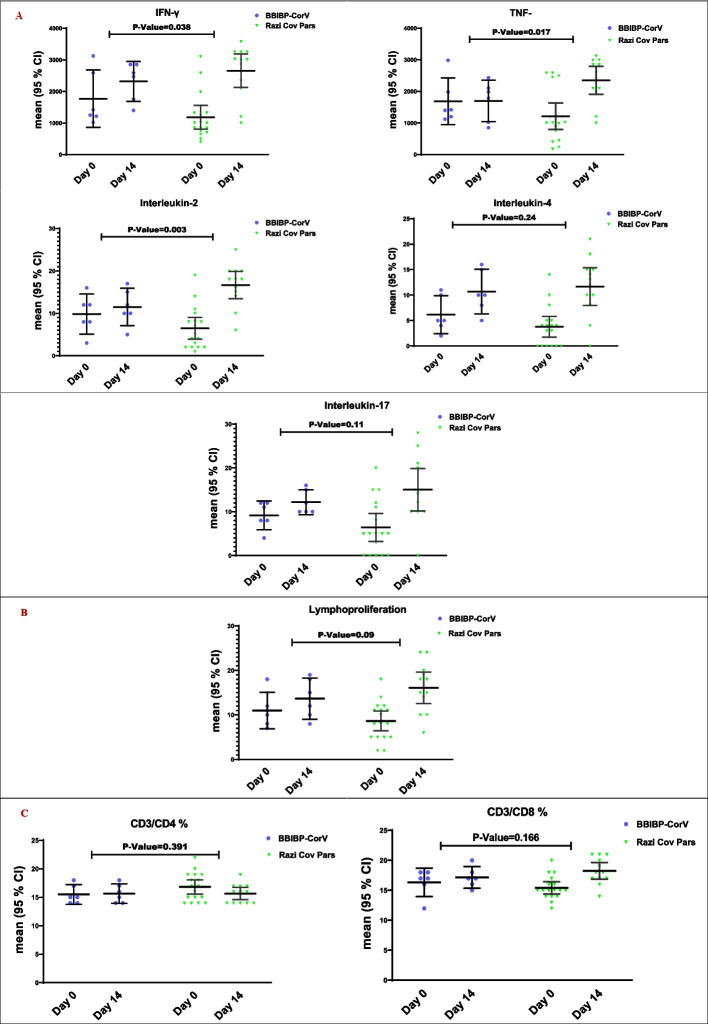
Comparison of the mean differences of the cell-mediated responses between days 0 and 14 in the BBIBP-CorV and RCP study groups in a subgroup of 18 participants. *P*-values for the *t*-test have been presented in the figures. Error bars are 95% CIs. **A** Specific cytokines were detected by ELISA (IFN-γ, TNF-α, interleukin 2, interleukin 4, interleukin 17), **B** lymphocyte proliferative potential response following stimulation by S antigen was measured by CFSE method, and **C** cell counts for lymphocytic subpopulations (CD3/CD4 ratio, CD3/CD8 ratio) using flow cytometry

Following stimulation with S antigen, IFN-γ, TNF-α, and IL-2 increased on day 14 compared with day 0 in both vaccine groups. Still, the response was statistically significantly higher in the RCP group than in BBIBP-CorV (*P*-value < 0.05) (Fig. 3A). Increase in IL-4, IL-17, and lymphocyte proliferation were seen in response to stimulation with S antigen in both vaccines, and the increase was higher (though not statistically significant) in the RCP vaccine than BBIBP-CorV (*P*-value > 0.05) (Fig. 3A, B). In flow cytometry, we observed a noticeable increase in the percentage of CD3 + /CD8 + in the RCP group (though it did not reach statistical significance), but it remained relatively unchanged in the BBIBP-CorV group (Fig. 3C). Overall, it seems that T helper 1 differentiation of T cells (increase in IFN-γ, IL-2, and percentage of CD3 + /CD8 +) is more marked in RCP vaccine recipients in response to stimulation with S antigen (see Fig. 3A, C).

#### Safety and reactogenicity

**Fig. 4:**
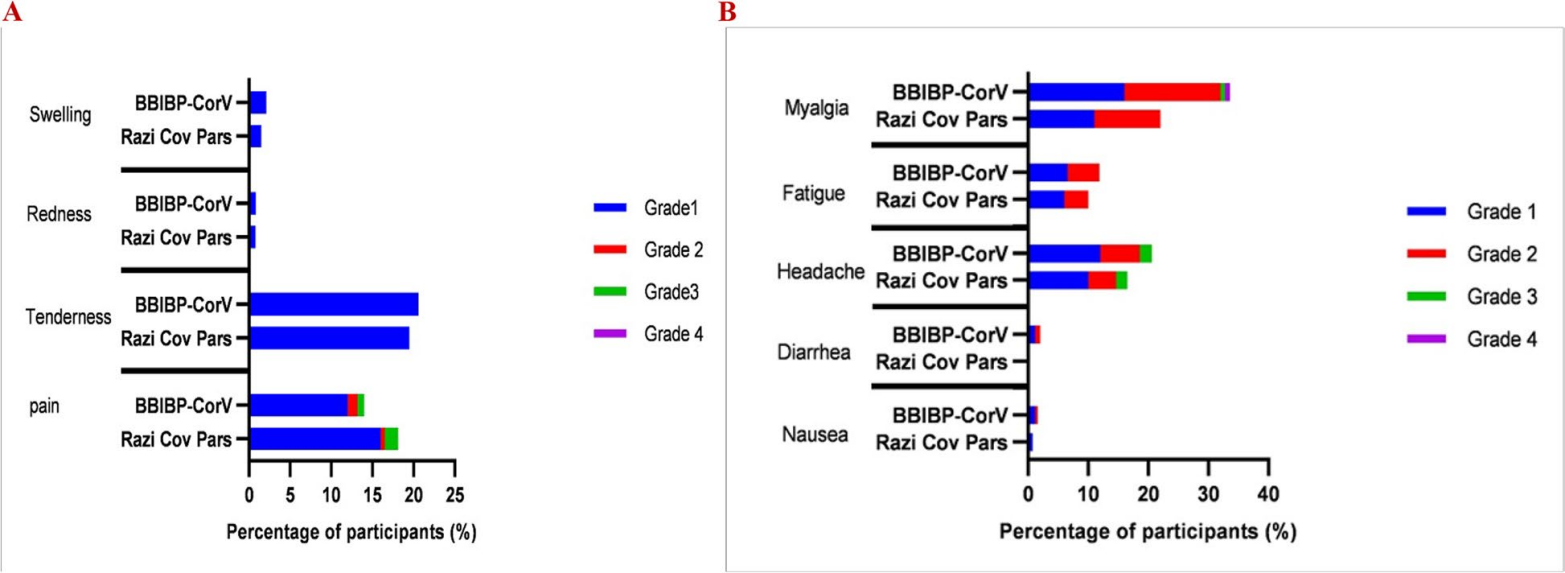
Local and systemic adverse reactions were reported within seven days after injection of RCP and BBIBP-CorV. Adverse reactions are graded according to the FDA toxicity grading scales. **A** Local adverse reactions. **B** Systemic adverse reactions

We did not observe any immediate allergic reaction in the study participants. The most common solicited local adverse reaction within the first-week post-vaccination was tenderness (20.6% in BBIBP-CorV and 19.5% in RCP groups). Other local reactions were pain, swelling, and redness (Fig. 4A). Grade III local adverse reactions were seen in 29 (12%) and 38 (16%) of BBIBP-CorV and RCP participants, all of which were fully recovered. The most prevalent solicited systemic adverse reaction within the first-week post-vaccination was myalgia (16% in BBIBP-CorV participants and 11% in RCP groups), followed by headache and fatigue (Fig. 4B). Grade III headache were seen in 12% of BBIBP-CorV and 10% of RCP participants. Two grade III and two grade IV cases of myalgia (0.8%) were seen only in the BBIBP-CorV group. No other grade III or IV solicited local and systemic adverse reaction was observed, and all of them were resolved during the follow-up period.

We identified 111 unsolicited adverse events (AE) during the month following the booster dose injection. All the cases received the necessary treatments and were followed until complete recovery (Additional file 1: Table S5). The rate of AEs occurrence was 6.52 (95% CI, 4.79–8.67) and 8.82 (95% CI, 6.79–11.26) per 1000 person-day in the RCP and BBIBP-CorV groups, and the difference was not statistically significant (Incidence rate ratio = 0.74, 95% CI 0.49, 1.09). Causality assessment identified seven unsolicited AEs with probable/suspected relationship to the study intervention, 4 in the RCP and 3 in the BBIBP-CorV groups (Additional file 1: Table S6). No vaccine-related or unrelated deaths were reported. In total, 2 cases of hospitalization were observed. The first one was a 62 years old woman diagnosed with myocardial infarction 15 days after receiving a BBIBP-CorV injection and discharged four days later. The other one is a 42 years old man that was admitted because of chest pain 12 days after receiving an RCP injection. The angiography was normal, and the patient was discharged 2 days later.

## Discussion

Our findings showed that neutralizing antibodies in RCP booster recipients increased 21 times the baseline after 2 weeks, indicating a robust boosting effect. This increase was about three times the baseline in the BBIBP-CorV booster recipients. The magnitude of neutralizing antibody response in the RCP group was about seven times higher than in the BBIBP-CorV group. We observed a similar pattern about the specific IgG antibodies against S1 and RBD antigens. The time interval between the booster dose and the primary vaccination did not affect the baseline antibody levels, and the differences were not statistically significant. We saw no immediate allergic vaccine reactions, and both groups’ self-limited solicited local and systemic reactions had similar frequencies. The occurrence of unsolicited adverse events over the one-month follow-up period did not differ significantly between the two groups.

We observed more robust antibody responses in the RCP vaccine booster dose recipients. Various explanations could be provided for this finding. First, BBIBP-CorV and RCP prime-boost combination is a heterologous boosting. Studies have reported that a heterologous boost offers a more robust immune response than a homologous boost [3, 12, 13, 18] and exposure to multiple spike variants broadens the neutralization [30, 31]. Second, BBIBP-CorV belongs to inactivated vaccine platform. Inactivated virus vaccines have a low spike protein compared to the total amount of virus protein content, and because some cleavage of S1 from S2 occurs during beta-propiolactone inactivation, they commonly present various and small amounts of spike protein to the immune system. Exposure of individuals primed with inactivated vaccines to a recombinant spike protein vaccine such as RCP with an enormous quantity of spike protein may trigger a particularly strong recall memory B cell response, causing a significant rise in neutralizing antibodies [18]. Third, the type of adjuvant used affects the antibody response. The use of adjuvants is common in order to boost the effectiveness of vaccines through mimicry of conserved molecules called pathogen-associated molecular patterns (PAMPs). Vaccine adjuvants enhance innate immune responses by stimulating dendritic cells, lymphocytes, and macrophages by imitating a natural infection [32]. RCP contains Razi Adjuvant System-01 (an oil-in-water emulsion), which may have contributed to a more significant antibody response in RCP booster dose recipients. Oil-in-water emulsion adjuvants could significantly reduce antigen doses and enhance the production of antigen-specific antibodies [32]. On the other hand, aluminum hydroxide adjuvant used in BBIBP-CorV results in limited T cell immunity due to alum’s tendency to attach to membranes instead of entering dendritic cells (DCs) and no intracellular transfer of antigens [33].

We observed slight differences in baseline antibody levels and post-booster antibody responses among the four tested groups with different prime-boosting intervals. Studies have shown that the level of antibodies starts to decline following the peak after primary vaccination and reaches critical levels after 3 to 6 months [34–36], so booster vaccination has been recommended after this period. General population vaccination against COVID-19 in Iran started in August and September 2021, about 3 to 4 months before the start of the current study. In addition, the timing of this study coincided with a surge in COVID-19 disease predominantly involving the Omicron strain in the Iranian population during the winter of 2022. Therefore, we had a high prevalence of COVID-19 disease and a subsequent high degree of wild virus circulation among the population in this period. Similar baseline antibody levels, regardless of the time of primary vaccination in our study, could be due to continuous exposure of the participants to various virus strains, albeit without clinical manifestation, following their first two doses of the BBIBP-CorV vaccine. This also explains similar post-booster antibody responses among the four tested groups with different prime-boosting intervals.

The incidence of adverse reactions was relatively low in RCP and BBIBP-CorV booster vaccinations, and most reported local and systemic adverse reactions were of grade I or II. The overall safety profile of RCP was similar to that of BBIBP-CorV boost, which was also comparable to the safety of the priming with two doses of BBIBP-CorV as reported previously [37]. Our safety data in the current study is consistent with data from other trials of homologous and heterologous third-dose boosters [12, 15, 18].

One of our study’s limitations was the short follow-up duration. Our study could be improved if all participants’ neutralizing antibody activity and antibody responses against S1 and RBD antigens were evaluated in months 3 and 6 after the booster dose, as it better reflects the effect of the booster vaccination. Furthermore, the coincidence of this study with a high prevalence of COVID-19 disease among the population may have contributed to the strengths of the observed immune responses. Measuring the humoral and cellular immune responses to booster doses in the absence of a concurrent COVID-19 outbreak could provide a more accurate assessment of the boosting ability of the vaccine. The few choices of vaccines available for primary vaccination within the national vaccination program also limited us. Making comparisons within a wider selection of prime and boost vaccines could improve our understanding of the population’s immune response to booster doses.

## Conclusions

In summary, this study showed that both RCP and BBIBP-CorV are safe and effective booster vaccines. RCP induced much more robust humoral and cellular immune responses than BBIBP-CorV, most likely due to its spike protein subunit platform and heterologous boosting characteristics in the current study participants.

### Supplementary Information

**Additional file 1: Table S1.** Geometric mean and 95% CI of specific antibody responses (AUC) to S1, RBD and Neutralizing antibody titer in the BBIBP-CorV and RCP groups over the predefined study time schedule. **Tables S2-S4.** Geometric mean, Geometric mean ratio, Geometric mean fold increase and Seroconversion and their 95% CI for Neutralizing antibodies, anti-RBD, and anti-S1 specific IgG antibodies in the BBIBP-CorV and Razi Cov Pars groups in the participants who received primary vaccination 3, 4, 5 and 6 month before booster dose over the predefined study time schedule. **Table S5.** Unsolicited adverse events with Not Related, Unlikely, Suspected/Possible, Probable and not assessable relationship to the BBIBP-CorV and Razi Cov Pars vaccines within one-month post-vaccination using ICD-10 code. Table S6. Unsolicited adverse events with probable/suspected relationship to the BBIBP-CorV and Razi Cov Pars vaccines using ICD-10 code. **Figure S1.** Gating strategy for CD3/CD4/CD8 and IFN-γ flow cytometry data analysis. **Figure S2**. Comparison of the baseline antibody levels and post-booster antibody responses among the four tested groups with different prime-boosting intervals (3, 4, 5 and 6 months before booster dose) on days 0 and 14.

## Data Availability

The data and materials that support the findings of this study are available from the corresponding author upon reasonable request.

## Abbreviations

AUC: Area under the curve
CD3: Cluster of differentiation 3
CD4: Clusters of differentiation 4
CD8: Clusters of differentiation 8
GM: Geometric mean
GMFI: Geometric mean fold increase
GMR: Geometric mean ratio
GMT: Geometric means titers
IFN-γ: Gamma interferon
IL-17: Interleukin 17
IL-2: Interleukin 2
IL-4: Interleukin 4
RBD: Receptor-binding domain
RCP: Razi Cov Pars vaccine
TNF-α: Tumor necrosis factor
