# Supplementary material for: Immunogenicity and safety of RAZI recombinant spike protein vaccine (RCP) as a booster dose after priming with BBIBP-CorV: a parallel two groups, randomized, double blind trial
Source: BMC Med. 2024 Feb 20;22:78. doi: 10.1186/s12916-024-03295-1 (PMC10877779; doi:10.1186/s12916-024-03295-1)
Supplement: Supplementary file 1 — Additional file 1: Table S1. Geometric mean and 95% CI of specific antibody responses (AUC) to S1, RBD and Neutralizing antibody titer in the BBIBP-CorV and RCP groups over the predefined study time schedule. Tables S2-S4. Geometric mean, Geometric mean ratio, Geometric mean fold increase and Seroconversion and their 95% CI for Neutralizing antibodies, anti-RBD, and anti-S1 specific IgG antibodies in the BBIBP-CorV and Razi Cov Pars groups in the participants who received primary vaccination 3, 4, 5 and 6 month before booster dose over the predefined study time schedule. Table S5. Unsolicited adverse events with Not Related, Unlikely, Suspected/Possible, Probable and not assessable relationship to the BBIBP-CorV and Razi Cov Pars vaccines within one-month post-vaccination using ICD-10 code. Table S6. Unsolicited adverse events with probable/suspected relationship to the BBIBP-CorV and Razi Cov Pars vaccines using ICD-10 code. Figure S1. Gating strategy for CD3/CD4/CD8 and IFN-γ flow cytometry data analysis. Figure S2. Comparison of the baseline antibody levels and post-booster antibody responses among the four tested groups with different prime-boosting intervals (3, 4, 5 and 6 months before booster dose) on days 0 and 14. [file 12916_2024_3295_MOESM1_ESM.docx]

Additional file 1

**Determination of immunogenicity**

**Enzyme-linked immunosorbent assays (ELISA) for evaluation of antibody titer**

The S1 and RBD-specific IgG antibody titer of the collected serum samples were evaluated through in-house ELISA. S1 and RBD antigens-coated plates (96-well high-binding; Greiner, Austria) were blocked with 3% of skimmed milk and washed. Then, the collected sera at a dilution of 1:100 up to 100000 were added to the plates in duplicates and incubated for 1 hour at 37° C. After three times washing, diluted anti-human IgG -HRP conjugates were added as secondary antibodies (Sigma, USA), followed by washing and detection with 3,5,3′,5′-tetramethylbenzidine (Millipore, USA). The OD value (450 nm) was read via Cytation 5 imaging multimode microplate reader (BioTek, USA). The results are expressed as area under curve (AUC) (21).

**Virus Neutralizing Test (VNT)**

The virus neutralization test was performed to evaluate the protective properties of RCP and BBIBP-Corv vaccines. SARS-CoV-2 (GISAID accession EPI_ISL_1398937) was isolated from the clinical human specimens and titrated (from 1 log to 11 log, in serial 1 log dilutions) to determine a 50% tissue culture infective dose (TCID50) through 96-well culture plates of VERO cells. Two-fold serial dilutions of heat-inactivated serums sample with the starting dilution of 1:4 (i.e., 1/4, 1/8, 1/16, 1/32, 1/64) were then mixed with an equal volume of 100 TCID50 of the SARS-CoV-2, and incubated at 37°C for 1 hour under 5% CO2. The pre-incubated SARS-CoV-2 was added to the VERO cells (4 ×10*5 cells/ml) in duplicate and incubated for ten days at 37°C under 5% CO2. After that, cytopathic effect (CPE) formation in wells was measured through the inverted optical microscope. The highest serum dilution that protected more than 50% of the cells from CPE was considered as the neutralization titer (21, 27).

**Evaluation of the Cellular Immune Response**

**Evaluation of lymphoproliferation by CFSE assay**

Cell proliferative ability of peripheral mononuclear cells (MNCs) was measured via Carboxy fluorescein succinimidyl ester (CFSE) (BioLegend, USA) proliferation assay, using a flow cytometer (BD FACS Lyric, USA). In brief, CFSE labeling was performed on separated MNCs, as the cells (1×10*6 cells in 1 ml PBS/ 2% FBS) and 1μL of 5 mM CFSE (the CFSE final concentration reached 1 μg/ml) were mixed in a 15-ml tube and rapidly inverted and vortexed for 10 seconds. After 15 minutes of incubation at 37°C in the dark and two times washing, the CFSE-stained MNCs were seeded in 96-well plates (1×10*5 cells in 100μl of RPMI with 10% FBS). The cells were stimulated with 5μl of PHA (GIBCO, USA) or S1 and S2 SARS-COV-2 protein (0·3μg/ml). After 72 h incubation in 37°C and 5% CO2, the cells were analyzed using a flow cytometer (BD FACS Lyric, USA) (21).

**Evaluation of the cell markers and intracellular IFN-γ of MNCs in vaccinated human**

To evaluate T lymphocyte differentiation, flow cytometric analyses of cell surface markers were performed on peripheral blood MNCs1. The cells were isolated with ficoll gradient centrifugation and cultured in stimulation with either S1 and S2 SARS-CoV-2 antigen (0.3 µg/ml) or phytohaemagglutinin (PHA) (Gibco, USA) in RPMI medium (Gibco, USA) with 5% fetal bovine serum (FBS) for 24 h at 37°C and 5% CO2. Then, the cells at the density of 10*6/100μL in staining buffer were stained with specific conjugated anti-CD3, CD4, and CD8 antibodies (APC-R700 anti-human CD3 clone SK7, BD; FITC mouse anti-human CD4 clone RPA-T4, BD Pharmingen; PE mouse anti-human CD8, BD Pharmingen) for 30 minutes at 4°C in the dark. Data acquisition was performed by BD FACS Lyric flow cytometer, and CD3, CD4, CD8, CD3/CD8, and CD3/CD4 cell counts were analyzed by FACSuite V 1.2.1 software.

Also, for intracellular IFN-γ cytokine staining, cells in culture were treated with Golgi Plug (BD, 2301kz) for four hours at 37°C, fixed with 250μl of paraformaldehyde (PFA) (BD Cell fix) for 20 minutes at RT. After washing, the cells permeabilized with 0.2% tween 20/ PBS for 15 minutes at RT and stained with APC conjugated anti IFN-γ antibody (BD) in 100μL of 0.1% tween 20 in PBS/FBS 2% (perm/wash buffer) for 30 minutes at four °C, then washed twice with perm/wash buffer and examined by BD FACS Lyric flow cytometer (21).

We analyzed the Flow cytometry data based on gating schematically represented in Figure S1. At first, the lymphocyte population was gated (R1 population), then the desired subpopulations such as CD3+, CD4+, or CD8+ and also double positive CD3+ CD8+ cells, and CD3+ CD4+ cells, also IFN-γ + cells were measured in R1 population.


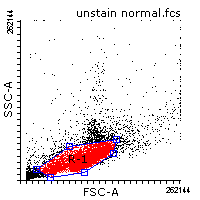

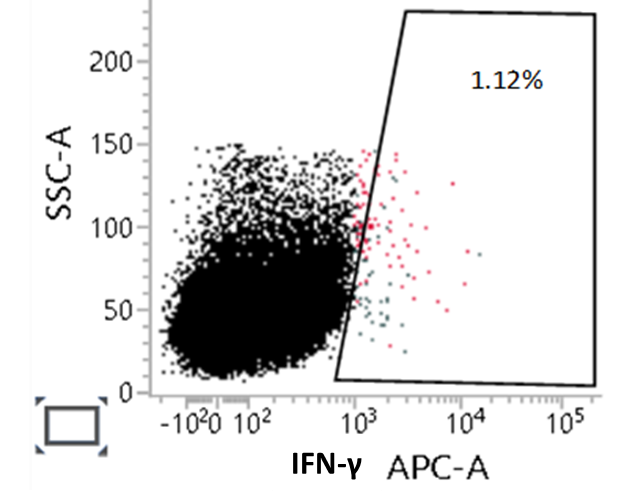


Figure S1. Gating strategy for CD3/CD4/CD8 and IFN-γ flow cytometry data analysis.

**Evaluation of the cytokine levels by ELISA**

Peripheral blood mononuclear cells were also assessed for secretion of specific cytokines after specific SARS-CoV-2 S1/S2 antigen (0.3 μg/ml) stimulation. The 72 h supernatant of cultured cells analyzed for the expression of Gamma interferon (IFN-γ), tumor necrosis factor (TNF-α), and interleukin IL-2, 4, 6, and 17 were detected by Enzyme-Linked Immunosorbent Assay (ELISA) (R&D, USA) according to the manufacturer’s instructions. The OD value (450 nm) was read by Cytation 5 imaging reader (BioTek, USA). The results are expressed as pg/ml (21).

Table S1- Geometric mean and 95% CI of specific antibody responses (AUC) to S1, RBD and Neutralizing antibody titer in the BBIBP-CorV and RCP groups over the predefined study time schedule.

|  | Neutralizing antibody titer^a^ | | Anti-SARS-CoV-2 S1 IgG level^b^ _AUC_^c^ | | Anti-SARS-CoV-2 RBD IgG level _AUC_ | |
| --- | --- | --- | --- | --- | --- | --- |
|  | BBIBP-CorV | RCP | BBIBP-CorV | RCP | BBIBP-CorV | RCP |
| GM^d^ (95% CI) | | | | | | |
| Baseline | 6 (5-8, n=239^*^) | 5 (4-7, n=239) | 72147 (59622-87304, n=239) | 66309.5 (54342.8-80911.4, n=239) | 58582.8 (48302.1-71051.7, n=239) | 52794.1 (43110.7-64652.6, n=238) |
| Day 14 | 18 (15-22, n=214) | 125 (109-143, n=203) | 328961 (284701-380102, n=204) | 1044273 (1005289-1084768, n=195) | 259265.1 (224307-299671.2, n=204) | 951747.6 (899100.7-1007477, n=195) |
| Day 90 | 312(223-437, n=59) | 552 (449-680, n=42) | 308456 (250278-380157, n=59) | 626882 (572052-686967, n=41) | 263928.3 (209107-333122, n=59) | 581952.1 (526995.3-642640.1, n=41) |
| Day 180 | 207 (154-278, n=48) | 491 (397-608, n=38) | 279847 (229168-341735, n=48) | 483199 (426863-546970, n=38) | 225948.7 (182647.2-279515.9, n=48) | 409590.2 (349022.3-480668.9, n=38) |

a were assessed via a conventional Virus Neutralization Test; b were measured using house ELISA kits and specific COVID-19 antigens (Native Antigen, UK); c Area Under the Curve; d Geometric Mean; e Geometric Mean Fold Increase; f Geometric Mean Ratio; g 4-fold increase compared to the baseline in neutralizing antibody titer or specific IgG antibody level (AUC); * The number of participants at each time point may be different due to spoilage of blood samples in the laboratory and withdrawal from the study.

|  | Neutralizing antibody titer^a^ | | | | | | | |
| --- | --- | --- | --- | --- | --- | --- | --- | --- |
|  | BBIBP-CorV | Razi Cov Pars | BBIBP-CorV | Razi Cov Pars | BBIBP-CorV | Razi Cov Pars | BBIBP-CorV | Razi Cov Pars |
|  | Received primary vaccination 2.5-3.5 months before booster dose | | Received primary vaccination 3.5-4.5 months before booster dose | | Received primary vaccination 4.5-5.5 months before booster dose | | Received primary vaccination 5.5-6.5 months before booster dose | |
| GM (95% CI)^d^ | | | | | | | | |
| Baseline | 5.48 (3.50-8.60, n=64) | 5.86 (3.66-9.37, n=64) | 7.29 (5.47-9.72, n=149) | 6.17 (4.56-8.34, n=147) | 6.86 (2.84-16-55, n=16) | 2.23 (0.87-5.69, n=15) | 2.80 (1.06-7.38, n=10) | 5.58 (1.63-19.04, n=13) |
| Day 14 | 17.12 (11.49-25.50, n=59) | 109.23 (91.12-130.95, n=56) | 18.30 (14.08-23.77, n=133) | 133.24 (111.79-158.80, n=122) | 25.67 (10.93-60.27, n=15) | 190.48 (83.71-433.46, n=13) | 19.17 (5.04-72.90, n=7) | 83.3 (31.39-220.94, n=12) |
| Day 90 | 308.53 (183.10-519.87, n=17) | 515.04 (312.68-848.36, n=13) | 340.79 (231.86-500.88, n=35) | 561.74 (440.03-717.11, n=27) | 171.07 (11.22-2607.15, n=6) | - | - | - |
| Day 180 | 170.53 (86.17-337.48, n=13) | 575.20 (413.88-799.40, n=11) | 235.56 (161.93-342.67, n=30) | 452.16 (338.68-603.67, n=25) | 158.10 (56.71-440.69, n=5) | - | - | - |
| GMFI (95% CI) ^e^ | | | | | | | | |
| Baseline | 1 (Reference) | 1 (Reference) | 1 (Reference) | 1 (Reference) | 1 (Reference) | 1 (Reference) | 1 (Reference) | 1 (Reference) |
| Day 14 | 3.06 (2.00-4.68) | 21.24 (13.20-34.20) | 2.41 (1.89-3.07) | 19.90 (13.78-28.73) | 3.73 (1.79-7.78) | 75.23 (26.11-216.80) | 8.75 (1.53-50.01) | 12.92 (3.18-52.38) |
| Day 90 | 51.86 (21.46-125.29) | 108.86 (27.64-428.67) | 41.70 (21.66-80.28) | 78.74 (37.73-164.32) | 63.42 (3.17-1268.00) | - | - | - |
| Day 180 | 22.98 (8.19-64.47) | 121.38 (32.79-449.29) | 24.78 (13.22-46.46) | 72.45 (34.96-150.14) | 97.87 (17.39-550.59) | - | - | - |
| GMR (95% CI) ^f^ | | | | | | | | |
| Baseline | 1 (Reference) | 1.07 (0.56-2.03) | 1 (Reference) | 0.84 (0.56-1.28) | 1 (Reference) | 0.32 (0.09-1.11) | 1 (Reference) | 1.99 (0.42-9.36) |
| Day 14 | 1 (Reference) | 6.38 (4.10-9.91) | 1 (Reference) | 7.28 (5.29-10.02) | 1 (Reference) | 7.42 (2.37-23.15) | 1 (Reference) | 4.34 (0.96-19.64) |
| Day 90 | 1 (Reference) | 1.67 (0.82-3.39) | 1 (Reference) | 1.65 (1.02-2.66) | 1 (Reference) | - | 1 (Reference) | - |
| Day 180 | 1 (Reference) | 3.37 (1.57-7.23) | 1 (Reference) | 1.92 (1.19-3.09) | 1 (Reference) | - | 1 (Reference) | - |
| Seroconversion , n/N (% )^g^ | | | | | | | | |
| Day 14 | 20/59 (34 %) | 46/56 (82 %) | 39/131 (30 %) | 90/121 (75 %( | 6/14 (42.86%) | 12/13 (92.3%) | 4/7 (57 %) | 9/12 (75 %) |
| Day 90 | 16/17 (94 % ) | 11/13 (84 %) | 33/35 (94 % ) | 26/27 (96 %( | 5/6 (83.3%) | - | - | - |
| Day 180 | 10/13(77%) | 10/11(91%) | 27/30(90%) | 25/25 (100%) | 5/5 (100%) | - | - | - |

Table S2 - Geometric mean, Geometric mean ratio, Geometric mean fold increase and Seroconversion and 95% CI of Neutralizing antibody titer in the BBIBP-CorV and Razi Cov Pars groups in the participants who received primary vaccination 3, 4, 5 and 6 month before booster dose over the predefined study time schedule.

a were assessed via a conventional Virus Neutralization Test; b were measured using house ELISA kits and specific COVID-19 antigens (Native Antigen, UK); c Area Under the Curve; d Geometric Mean; e Geometric Mean Fold Increase; f Geometric Mean Ratio; g 4-fold increase compared to the baseline in neutralizing antibody titer or specific IgG antibody level (AUC); * The number of participants at each time point may be different due to spoilage of blood samples in the laboratory and withdrawal from the study.

|  | Anti-SARS-CoV-2 S1 IgG level^b^ _AUC_^c^ | | | | | | | |
| --- | --- | --- | --- | --- | --- | --- | --- | --- |
|  | BBIBP-CorV | Razi Cov Pars | BBIBP-CorV | Razi Cov Pars | BBIBP-CorV | Razi Cov Pars | BBIBP-CorV | Razi Cov Pars |
|  | Received primary vaccination 2.5-3.5 months before booster dose | | Received primary vaccination 3.5-4.5 months before booster dose | | Received primary vaccination 4.5-5.5 months before booster dose | | Received primary vaccination 5.5-6.5 months before booster dose | |
| GM (95% CI)^d^ | | | | | | | | |
| Baseline | 62193.0 (43824.1-88261.3, n=63) | 52542.7 (37715.8-73198.3, n=64) | 81123.4 (63463.5-103697.7, n=150) | 78023.8 (60005.2-101453, n=147) | 72297.1 (30094.6-173681.2, n=16) | 39642.4 (14754.1-106514.2, n=15) | 31559.6 (11705.7-85087.6, n=10) | 59980.4 (21476.7-167513.6, n=13) |
| Day 14 | 318452.2 (239347.0-423702.1, n=59) | 1025577.0 (953730.1-1102836.0, n=56) | 338102.2 (282356.6-404853.6, n=125) | 1051620.0 (1001360.0-1104403.0, n=116) | 334362.1 (164890.7-678012.9, n=13) | 1104324.0 (1004686.0-1213843.0, n=11) | 257232.2 (92576.5-714742.8, n=7) | 1008636.0 (791194.3-1285837.0, n=12) |
| Day 90 | 246173.9 (148618.8-407765.5, n=17) | 597988.5 (507891.5-704068.1, n=13) | 335660.8 (261618.1-430658.8, n=35) | 632516.8 (558478.8-716370.2, n=26) | 310639.0 (155174.0-621860.3, n=6) | - | - | - |
| Day 180 | 281444.7 (182497.4-434039.7, n=13) | 453338.9 (340897.7- 602867.6, n=12) | 281688.5 (216074.6-367227, n=30) | 492137.3 (426384.5-568029.8, n=25) | 265101.8 (125790.4-558698.9, n=5) | - | - | - |
| GMFI (95% CI)^e^ | | | | | | | | |
| Baseline | 1 (Reference) | 1 (Reference) | 1 (Reference) | 1 (Reference) | 1 (Reference) | 1 (Reference) | 1 (Reference) | 1 (Reference) |
| Day 14 | 5.57 (4.13-7.54) | 19.61 (13.95-27.57) | 3.84 (3.02-4.90) | 13.83 (10.43-18.35) | 4.39 (1.79-10.76) | 20.61 (5.54-76.72) | 7.47 (1.15-48.46) | 14.14 (5.49-36.42) |
| Day 90 | 4.67 (2.27-9.62) | 11.22 (5.11-24.66) | 4.33 (2.49-7.52) | 6.68 (3.41-13.11) | 7.26 (1.23-43.01) | - | - | - |
| Day 180 | 3.70 (1.75-7.82) | 8.15 (3.32-20.03) | 3.74 (2.10-6.68) | 6.66 (3.20-13.85) | 10.99 (2.32-52.03) | - | - | - |
| GMR (95% CI)^f^ | | | | | | | | |
| Baseline | 1 (Reference) | 0.84 (0.52-1.36) | 1 (Reference) | 0.96 (0.67-1.38) | 1 (Reference) | 0.55 (0.16-1.93) | 1 (Reference) | 1.90 (0.48-7.52) |
| Day 14 | 1 (Reference) | 3.22 (2.39-4.34) | 1 (Reference) | 3.11 (2.57-3.77) | 1 (Reference) | 3.30 (1.58-6.91) | 1 (Reference) | 3.92 (1.89-8.12) |
| Day 90 | 1 (Reference) | 2.43 (1.37-4.32) | 1 (Reference) | 1.88 (1.39-2.55) | 1 (Reference) | - | - | - |
| Day 180 | 1 (Reference) | 1.61 (.97-2.65) | 1 (Reference) | 1.75 (1.27-2.39) | - | - | - | - |
| Seroconversion , n/N (% )^g^ | | | | | | | | |
| Day 14 | 33/58 (56 %) | 51/56 (91%) | 56/125 (45 %) | 84/115 (73%) | 7/12 (58 %) | 8/11 (72 %) | 3/7 (43 %) | 8/12 (67 %) |
| Day 90 | 9/17 (52.9%) | 9/13 (69 %) | 15/35 (43%) | 13/25 (52 %) | 4/6 (66 %) | - | - | - |
| Day 180 | 8/13(61.5%) | 8/12 (66.6%) | 12/30 (40%) | 15/25 (60%) | 4/5 (80%) | - | - | - |

Table S3- Geometric mean, Geometric mean ratio, Geometric mean fold increase and Seroconversion and 95% CI of Specific IgG antibody response against Sars-Cov-2 spike protein antigens S1 in the BBIBP-CorV and Razi Cov Pars groups in the participants who received primary vaccination 3, 4, 5 and 6 month before booster dose over the predefined study time schedule.

a were assessed via a conventional Virus Neutralization Test; b were measured using house ELISA kits and specific COVID-19 antigens (Native Antigen, UK); c Area Under the Curve; d Geometric Mean; e Geometric Mean Fold Increase; f Geometric Mean Ratio; g 4-fold increase compared to the baseline in neutralizing antibody titer or specific IgG antibody level (AUC); * The number of participants at each time point may be different due to spoilage of blood samples in the laboratory and withdrawal from the study.

|  | Anti-SARS-CoV-2 RBD IgG level^b^ _AUC_^c^ | | | | | | | |
| --- | --- | --- | --- | --- | --- | --- | --- | --- |
|  | BBIBP-CorV | Razi Cov Pars | BBIBP-CorV | Razi Cov Pars | BBIBP-CorV | Razi Cov Pars | BBIBP-CorV | Razi Cov Pars |
|  | Received primary vaccination 2.5-3.5 months before booster dose | | Received primary vaccination 3.5-4.5 months before booster dose | | Received primary vaccination 4.5-5.5 months before booster dose | | Received primary vaccination 5.5-6.5 months before booster dose | |
| GM (95% CI)^d^ | | | | | | | | |
| Baseline | 49485.23 (34852.67-70261.13, n=63) | 42639.24 (30499.7-59610.57, n=64) | 66359.87 (51719.62-85144.35, n=150) | 61567.47 (47014.94-80624.45, n=147) | 59112.5 (24565.5-142243.9, n=16) | 32830.5 (12311.5-87547.9, n=15) | 25777.3 (9258.7-71766.8, n=10) | 45963.6 (16394.0-128867.3, n=13) |
| Day 14 | 248651.7 (188470.7-328049.4, n=59) | 921411 (850382.7-998371.9, n=56) | 266004.6 (221835.7-318967.9, n=125) | 974266.1 (912295.4-1040446, n=116) | 271233.2 (125512.4-586136.7, n=13) | 970087.5 (901957.4-1043364.0, n=11) | 214449.1 (76539.5-600845.9, n=7) | 867767.0 (452842.1-1662875.0, n=12) |
| Day 90 | 209870.1 (117836.1-373785.6, n=17) | 562049.9 (466647.3-676956.9, n=13) | 290837.1 (222325.9-380460.4, n=35) | 585398.7 (512043-669263.4, n=26) | 246779.9 (105474.5-577393.6, n=6) | - | - | - |
| Day 180 | 217340.9 (134523.7-351143.1, n=13) | 362928.4 ( 257337.2-511846.3, n=12 ) | 231647.5 (174812.3-306960.9, n=30) | 427869.9 (353102.2-518469.4, n=25) | 21528.7 (111035.1-417312.1, n=5) | - | - | - |
| GMFI (95% CI)^e^ | | | | | | | | |
| Baseline | 1 (Reference) | 1 (Reference) | 1 (Reference) | 1 (Reference) | 1 (Reference) | 1 (Reference) | 1 (Reference) | 1 (Reference) |
| Day 14 | 5.47 (4.05-7.40) | 21.72 (15.40-30.66) | 3.76 (2.93-4.84) | 16.64 (12.38-22.37) | 4.37 (1.71-11.10) | 22.2 (11.33-43.35) | 7.8 (1.17-52.16) | 16.0 (5.64-45.62) |
| Day 90 | 5.01 (2.44-10.31) | 13.49 (5.92-30.77) | 4.88 (2.79-8.56) | 7.25 (3.67-14.29) | 6.83 (1.16-40.06) | - | - | - |
| Day 180 | 3.71 (1.69-8.14) | 8.35 (3.14-22.20) | 4.02 (2.25-7.18) | 6.82 (2.26-14.28) | 10.64 (2.39-47.35) | - | - | - |
| GMR (95% CI) ^f^ | | | | | | | | |
| Baseline | 1 (Reference) | 0.86 (0.53-1.39) | 1 (Reference) | 0.92 (0.64-1.33) | 1 (Reference) | 0.56 (0.16-1.95) | 1 (Reference) | 1.78 (0.44-7.19) |
| Day 14 | 1 (Reference) | 3.71 (2.77-4.96) | 1 (Reference) | 3.66 (3.00-4.46) | 1 (Reference) | 3.58 (1.60-7.98) | 1 (Reference) | 4.05 (1.40-11.68) |
| Day 90 | 1 (Reference) | 2.68 (1.39-5.17) | 1 (Reference) | 2.01 (1.45-2.79) | 1 (Reference) | - | - | - |
| Day 180 | 1 (Reference) | 1.66 (0.94-2.94) | 1 (Reference) | 1.84 (1.30-2.61) | - | - | - | - |
| Seroconversion , n/N (%I)^g^ | | | | | | | | |
| Day 14 | 34/58 (56 %) | 51/56 [91%) | 56/125 (44.8%) | 88/116 (75.8%) | 7/12 (58 %) | 8/11 (73 %) | 3/7 (43 %) | 10/12 (83 %) |
| Day 90 | 10/17 (58 %) | 9/13 (69 %) | 17/35 (48.5%) | 14/26 (53.8%) | 4/6 (66.6%) | - | - | - |
| Day 180 | 8/13 (61.5%) | 8/12 (66.8%) | 13/30 (43.3%) | 14/25 (56%) | 4/5 (80%) | - | - | - |

Table S4- Geometric mean, Geometric mean ratio, Geometric mean fold increase and Seroconversion and 95% CI of Specific IgG antibody response against Sars-Cov-2 spike protein antigens RBD in the BBIBP-CorV and Razi Cov Pars groups in the participants who received primary vaccination 3, 4, 5 and 6 month before booster dose over the predefined study time schedule.

a were assessed via a conventional Virus Neutralization Test; b were measured using house ELISA kits and specific COVID-19 antigens (Native Antigen, UK); c Area Under the Curve; d Geometric Mean; e Geometric Mean Fold Increase; f Geometric Mean Ratio; g 4-fold increase compared to the baseline in neutralizing antibody titer or specific IgG antibody level (AUC); * The number of participants at each time point may be different due to spoilage of blood samples in the laboratory and withdrawal from the study.

Table S5- Unsolicited adverse events with Not Related, Unlikely, Suspected/Possible, Probable and not assessable relationship to the BBIBP-CorV and Razi Cov Pars vaccines within one-month post-vaccination using ICD-10 code.

| ICD code | ICD Description | BBIBP-CorV | Razi Cov Pars | Total |
| --- | --- | --- | --- | --- |
| B34.9 | Viral infection, unspecified | 13 | 5 | 18 |
| I10 | Essential (primary) hypertension | 9 | 4 | 13 |
| M79.6 | Pain in limb | 3 | 5 | 8 |
| R51 | Headache | 2 | 3 | 5 |
| M19.9 | Arthrosis, unspecified | 2 | 2 | 4 |
| R53 | Malaise and fatigue | 0 | 3 | 3 |
| U07.1 | COVID-19, virus identified | 5 | 3 | 8 |
| U07.2 | COVID-19, virus not identified | 2 | 1 | 3 |
| I25.1 | Atherosclerotic heart disease | 1 | 1 | 2 |
| J02 | Acute pharyngitis | 0 | 2 | 2 |
| K29 | Gastritis and duodenitis | 2 | 0 | 2 |
| R03.0 | Elevated blood-pressure reading, without diagnosis of hypertension | 1 | 1 | 2 |
| R07.4 | Chest pain, unspecified | 1 | 1 | 2 |
| R10 | Abdominal and pelvic pain | 2 | 0 | 2 |
| R42 | Dizziness and giddiness | 1 | 1 | 2 |
| A09.9 | Gastroenteritis and colitis of unspecified origin | 1 | 0 | 1 |
| F41 | Other anxiety disorders | 1 | 0 | 1 |
| F41.9 | Anxiety disorder, unspecified | 1 | 0 | 1 |
| F44 | Dissociative [conversion] disorders | 1 | 0 | 1 |
| G43 | Migraine | 1 | 0 | 1 |
| H66.9 | Otitis media, unspecified | 0 | 1 | 1 |
| H81.1 | Benign paroxysmal vertigo | 1 | 0 | 1 |
| J00 | Acute nasopharyngitis [common cold] | 0 | 1 | 1 |
| K40 | Inguinal hernia | 1 | 0 | 1 |
| L03 | Cellulitis | 1 | 0 | 1 |
| L08.9 | Local infection of skin and subcutaneous tissue, unspecified | 1 | 0 | 1 |
| L50.9 | Urticaria, unspecified | 1 | 0 | 1 |
| M51 | Other intervertebral disc disorders | 0 | 1 | 1 |
| M54.1 | Radiculopathy | 1 | 0 | 1 |
| M54.5 | Low back pain | 1 | 0 | 1 |
| N32.9 | Bladder disorder, unspecified | 1 | 0 | 1 |
| N92.6 | Irregular menstruation, unspecified | 0 | 1 | 1 |
| R04.0 | Epistaxis | 0 | 1 | 1 |
| R05 | Cough | 1 | 0 | 1 |
| R06.0 | Dyspnoea | 1 | 0 | 1 |
| R07.1 | Chest pain on breathing | 1 | 0 | 1 |
| R20.2 | Paraesthesia of skin | 0 | 1 | 1 |
| R25.2 | Cramp and spasm | 1 | 0 | 1 |
| R30.0 | Dysuria | 1 | 0 | 1 |
| loss to follow up | ---- | 0 | 5 | 6 |
| Routine followup | ---- | 2 | 4 | 6 |
| Total |  | 64 | 47 | 111 |

Table S6- Unsolicited adverse events with probable/suspected relationship to the BBIBP-CorV and Razi Cov Pars vaccines using ICD-10 code.

| ICD code | final ICD | BBIBP-CorV | Razi Cov Pars | Total |
| --- | --- | --- | --- | --- |
| L50.9 | Urticaria, unspecified | 1 | 1 | 2 |
| R42 | Dizziness and giddiness | 1 | 1 | 2 |
| K12 | Recurrent oral aphthae | 0 | 1 | 1 |
| R25.2 | Cramp and spasm | 1 | 0 | 1 |
| R53 | Malaise and fatigue | 0 | 1 | 1 |
| Total |  | 3 | 4 | 7 |

Figure S2- Comparison of the baseline antibody levels and post-booster antibody responses among the four tested groups with different prime-boosting intervals (3, 4, 5 and 6 months before booster dose) on days 0 and 14.

P-values reflect 2-tailed ANOVA tests.
